# Evaluation of a machine learning system for genomic antimicrobial susceptibility determination on a clinically representative test set

**DOI:** 10.1128/spectrum.00564-25

**Published:** 2026-01-27

**Authors:** Jason D. Wittenbach, Arolyn Conwill, Hayden Sansum, Alison Gassett, Adam Gardner, Allison Brookhart, Talia Hollowell, Paul Knysh, Nicholas B. Worley, Nicole Billings, Ian C. Herriott, Julie A. Shimabukuro, Kathleen A. Quan, Keith M. Madey, Susan S. Huang, Mohamad R. A. Sater, Cassiana E. Bittencourt, Miriam H. Huntley

**Affiliations:** 1 Day Zero Diagnostics, Inc, Watertown, Massachusetts, USA; 2 Department of Pathology and Laboratory Medicine, University of California Irvine Health, Orange, California, USA; 3 Epidemiology and Infection Prevention, University of California Irvine Health 150365 , Irvine, California, USA; The Ohio State University, Columbus, Ohio, USA

**Keywords:** antimicrobial resistance, machine learning, microbial genomics, NGS

## Abstract

Next-generation sequencing is anticipated to transform infectious disease treatment by enabling faster diagnosis, yet a clinical-grade method for genomic AST determination is still lacking. We assessed the accuracy of Keynome *g*AST, a *k*-mer based machine learning system for genomic AST, on 956 clinical bacterial isolates. We compared 7,801 predictions of susceptible (S), intermediate (I), and resistant (R) across 97 species-drug combinations against phenotypic AST results. Across the full data set, the Keynome *g*AST Qualified panel achieved a categorical agreement of 96.9% with very major and major error rates of 1.4% and 0.8%, respectively. At the level of individual species-drug combinations with sufficient data to assess, performance was comparable to the aggregate, with median categorical agreement of 97.4%. We also compared the performance of Keynome *g*AST to ResFinder, a simple resistance marker approach, in distinguishing susceptible vs non-susceptible samples. On the full data set, Keynome *g*AST performance was significantly better than that of ResFinder (binary accuracy of 96.0% vs 83.5%). When analyzed at the species-drug level, ResFinder’s poor performance was seen to be driven by either low sensitivity due to phenotypically resistant samples lacking resistance markers (18.0% of such samples) or low specificity due to the presence of resistance markers in phenotypically susceptible samples (15.1% of such samples). In total, these results demonstrate the dual advantages of machine learning and whole-genome profiling that Keynome *g*AST has over simple resistance marker approaches, enabling high accuracy genomic AST across a range of clinically relevant species-drug combinations.

**IMPORTANCE:**

Microbial genome sequencing presents an exciting opportunity for rapid diagnosis of infectious diseases, but interpreting the resulting data for clinical use remains a challenge. We report a new machine learning method that predicts a bacterial strain's antibiotic resistance profile based solely on its genomic sequence. This method could lead to new, faster diagnostic tools that quickly identify the most effective antibiotic therapy.

## INTRODUCTION

Using next-generation sequencing (NGS) of microbial genomes to infer the antimicrobial susceptibility test (AST) profile of an infecting pathogen offers a powerful opportunity to improve current infectious disease diagnostics. While the potential for NGS to provide faster results than current standard-of-care AST diagnostics and to thereby reduce the time to appropriate antibiotics has been recognized for over a decade (1, 2), there are currently no FDA approved NGS-based AST diagnostics. A major obstacle for clinical-grade diagnostics is the algorithmic challenge of genomic AST: reliably determining the phenotypic antimicrobial resistance pattern from the microbial genomic data for a comprehensive set of species and drugs. Traditional bioinformatics methods for performing genomic AST rely almost exclusively on the detection of known resistance markers annotated in published resistance marker databases (3–6), such that the simple presence of any markers in a pathogen genome predicts a non-susceptible phenotype. These databases are limited both by the extent and depth of investigation that has been conducted by the scientific community, often using focal data sets, and the challenge of hand curation of the results into a structured, standardized database (7). Moreover, such resistance marker approaches fall short in cases of complex multi-locus mechanisms where determining the presence or absence of a resistance marker is insufficient. They can provide false negative results if the database lacks determinants of resistance that are currently unknown or uncharacterized and, conversely, can provide false positive results if a resistance gene is present but not expressed or is insufficient to confer resistance alone. Lastly, they cannot distinguish between intermediate and resistant AST phenotypes, limiting their applicability in clinical contexts.

Machine learning (ML) methods offer a promising, conceptually distinct alternative to traditional genomic AST determination (8). In this approach, an algorithm is trained on a data set comprising the whole genome sequencing (WGS) data of clinically relevant pathogens matched to their phenotypically derived AST results to learn the genomic associations predictive of resistance and susceptibility. The use of ML to perform genomic AST presents multiple advantages over the traditional resistance marker approach: (1) the model can discover regions in the genome that are predictive of AST rather than being constrained to only previously annotated markers; (2) complex, nonlinear interactions can be captured by the model where simple presence/absence is insufficient, and (3) the model can predict susceptible (S), intermediate (I), and resistant (R) phenotypes using a large-scale data-driven approach to learn how to distinguish between signatures associated with each phenotype from the entirety of the genome. Various ML methods for genomic AST have been developed to date (9–13) though most do not distinguish between intermediate and resistant phenotypes.

In this study, we assessed Keynome *g*AST (genomic AST), an ML system that predicts S, I, and R AST phenotypes from pathogen WGS data. We trained Keynome *g*AST on a large-scale data set containing the WGS data of >40,000 bacterial isolates paired with their corresponding phenotypic AST results. We evaluated Keynome *g*AST accuracy on a separate test set of nearly 1,000 clinical bacterial isolates from the UC Irvine Medical Center collected over 3 months, comparing Keynome *g*AST predictions to the phenotypic AST results from routine care in the hospital lab. We further compared Keynome *g*AST performance to a traditional resistance marker approach (ResFinder).

## MATERIALS AND METHODS

### Isolate collection, phenotyping, and WGS

The University of California Irvine (UCI) Microbiology Lab collected a total of 1,536 clinical bacterial isolates previously processed for routine clinical care from patients treated in the Emergency Department or inpatient units between 4/20/2023-8/12/2023. In the final test data set (see *Test Set Data Exclusion* below), 39.5% of isolates were from patients seen in the Emergency Department, 23.5% from surgical inpatient units, 7.1% non-surgical ICU units, and 29.3% from various other units (oncology, labor and delivery, neuro, etc). The UCI Microbiology Lab performed phenotypic testing using routine procedures. Species identification was performed using MALDI-TOF (Vitek MS v3 with MYLA v4.5, bioMérieux, Marcy l'Etoile, France) or Vitek 2 (bioMérieux) if MALDI-TOF was unable to identify the isolate. Phenotypic AST was performed using Vitek 2 (bioMérieux), except for *Streptococcus agalactiae* and *Streptococcus pneumoniae* which were tested by Kirby-Bauer (Becton Dickinson, Sparks, MD), Etest (bioMérieux), and MTS (Liofilchem, Roseto degli Abruzzi, TE, Italy). Day Zero Diagnostics (DZD) re-interpreted all raw Minimum Inhibitory Concentration (MIC)/disk diameter values to S/I/R categories using CLSI M100 (32nd edition) where possible. If there were multiple breakpoints for different indications, parenteral breakpoints or those most relevant to bloodstream infections were preferentially selected.

Isolates were collected from purity or subculture plates and then shipped to DZD, where they were processed for short-read WGS. Genomic DNA was isolated using organic aqueous-phase, bead-beating, crude extraction followed by a QIAamp 96 DNA QIAcube HT Kit (Qiagen, Venlo, Netherlands) purification. Sequencing libraries were prepared with Nextera tagmentation (Illumina, San Diego, CA) using unique, dual-indexed primers and 2 × 150 bp paired-end reads sequencing was performed on Illumina HiSeqX or NovaSeqX by the commercial provider Psomagen (Rockville, MD).

### Bioinformatics processing

Illumina paired end sequencing data underwent quality and adapter trimming using Trimmomatic v0.38. Taxonomic identification was performed using Kraken v1.1.1 and a custom reference database. *K*-mers (subsequences of integer length *k*) of length 20 were derived from sequencing reads and used for clonality assessment and for generating machine learning model features. A depth-based analysis was used to exclude low-confidence *k*-mers (e.g., sequencing errors) and *k*-mer presence/absence vectors were generated for each sample. Genome assemblies were generated independently from trimmed reads using shovill (14). Multi-Locus sequence typing (MLST) from assemblies was determined with ARIBA v2.14.6 and MLST definitions obtained from PubMLST (15).

To provide a comparison to traditional resistance marker detection strategies, the tool ResFinder (ResFinder/PointFinder v4.3.0; ResFinder database v2.1.0; PointFinder mutation database v3.0.0) was used to identify resistance markers in genome assemblies and to infer resistance phenotype (3). Default parameters were used for both ResFinder and PointFinder; PointFinder output was merged with ResFinder output via a custom script. ResFinder’s built-in logic predicts “Resistant” for a drug if a relevant gene or point mutation was detected (which we interpreted as non-susceptible), otherwise “Not Resistant” (which we interpreted as susceptible). ResFinder’s database is species-agnostic, as are its phenotypic predictions based on presence/absence genes. The PointFinder point mutation databases are taxa-specific, including *E. faecalis*, *E. faecium, E. coli*, *Klebsiella*, and *S. aureus*. An antibiotic was considered covered by ResFinder/PointFinder if it or its drug class (e.g., quinolone) was included at least once in database annotations. 14 of the 97 species-drug combinations analyzed by Keynome *g*AST in this data set were considered not covered by ResFinder, corresponding to the drugs trimethoprim/sulfamethoxazole and ampicillin/sulbactam.

### Test set data exclusion

Isolates were excluded if the phenotypic AST or genomic sequencing data did not meet quality control (QC) criteria, including failed sequencing data checks for species identity and contamination, sequencing quality, and assembly quality (see Supplementary Methods). Isolates from the test data set that were determined to be clonal with any from the training data set, identified based on genomic relatedness (measured using a *k*-mer based similarity method; Supplementary Methods), were also excluded. Finally, isolates that lacked relevant phenotypic AST for any species-drug combinations on the Keynome *g*AST panels were also excluded from the test data set. A total of 956 isolates passed all QC metrics and were used in the remainder of the study.

### Machine learning methods

The Keynome *g*AST system is a set of machine learning models, one for each relevant species-drug combination, that are trained to learn microbial genomic features predictive of AST phenotype in an unbiased fashion. Briefly, these supervised learning models are trained to predict AST results—in the form of susceptible (S), intermediate (I), or resistant (R) categories—from short read WGS sequencing data inputs featurized via *k*-mers. The model for each species-drug combination assessed was assigned to either the “Qualified” or “R&D Stage” panel based on an internal assessment of model performance. More detailed information on training data set, model architecture, training procedure, and panel definitions can be found in the Supplementary Methods.

Based on these criteria, the Keynome *g*AST (v0.7.5.0) release used in this study comprised 70 species-drug combinations in the Qualified panel and 74 species-drug combinations in the R&D Stage panel. Insufficient training data set size was a significant factor limiting model accuracy and preventing their inclusion in the Qualified panel; >80% of R&D Stage models had <500 resistant or susceptible samples in the training data set.

### Data analysis

Keynome *g*AST predictions were assessed wherever both predictions and phenotypes were available. Though 144 species-drug combinations were available from both Keynome *g*AST panels, only those with at least one isolate in the test data set with a relevant phenotypic AST result were assessed. Conversely, any phenotypic AST results without matching Keynome *g*AST predictions were not evaluated.

Accuracy metrics were computed both in aggregate, combining all predictions across all species-drug combinations, and individually, for each species-drug combination tested. Genomic AST predictions were compared to phenotypic AST measurements to assess accuracy, using the following metrics:

- Categorical agreement (CA): Percent of genomic AST predictions that agreed with the phenotypic AST results (in terms of interpretive category: S/I/R).
- Very major error (vME) rate: Percent of phenotypically R cases with an incorrect genomic AST prediction of S.
- Major error (ME) rate: Percent of phenotypically S cases with an incorrect genomic AST prediction of R.
- Minor error (mE) rate: Percent of cases with an incorrect genomic AST prediction where either the phenotypic AST or the genomic AST prediction is I.

For an analysis of resistance marker-based predictions and direct comparison to those from Keynome *g*AST, intermediate and resistant ground truth phenotypes and predictions were grouped into a joint non-susceptible (NS) category, and performance metrics were calculated as follows:

- Binary accuracy: Percent of genomic AST predictions that agreed with the phenotypic AST results (at the level of binary prediction: S/NS)
- Sensitivity: Percent of phenotypically NS cases with a correct genomic AST prediction of NS.
- Specificity: Percent of phenotypically S cases with a correct genomic AST prediction of S.
- Positive predictive value (PPV): Percent of cases with a genomic AST prediction of NS that are phenotypically NS.
- Negative predictive value (NPV): Percent of cases with a genomic AST prediction of S that are phenotypically S.

Positive predictive value (PPV) was also calculated for each individual resistance marker and marker combination detected in a given species-drug data set, restricted to those found in at least 10 isolates across the entire data set. PPV is equal to the percent of samples in which the marker was detected that are phenotypically NS.

Binomial proportion confidence intervals (*CIs*, Wilson score interval) for all the above metrics are reported at the 95% confidence level. Statistical significance of performance differences between Keynome *g*AST and ResFinder was evaluated using McNemar’s test with false discovery rate correction for each metric analyzed (Benjamini-Hochberg correction, *α* = 0.05). A two-tailed binomial test (*α* = 0.05) was used to determine if a given CA or ME rate was statistically above/below and the corresponding FDA thresholds of 90%/3%, respectively. For the vME rate, the FDA criteria directly specify an upper bound on the upper/lower 95% confidence interval bounds of 1.5%/7.5%. The 95% vME rate confidence interval was compared directly to these bounds, and the test was deemed inconclusive if the measured confidence interval contained the target FDA confidence interval.

## RESULTS

**Fig 1:**
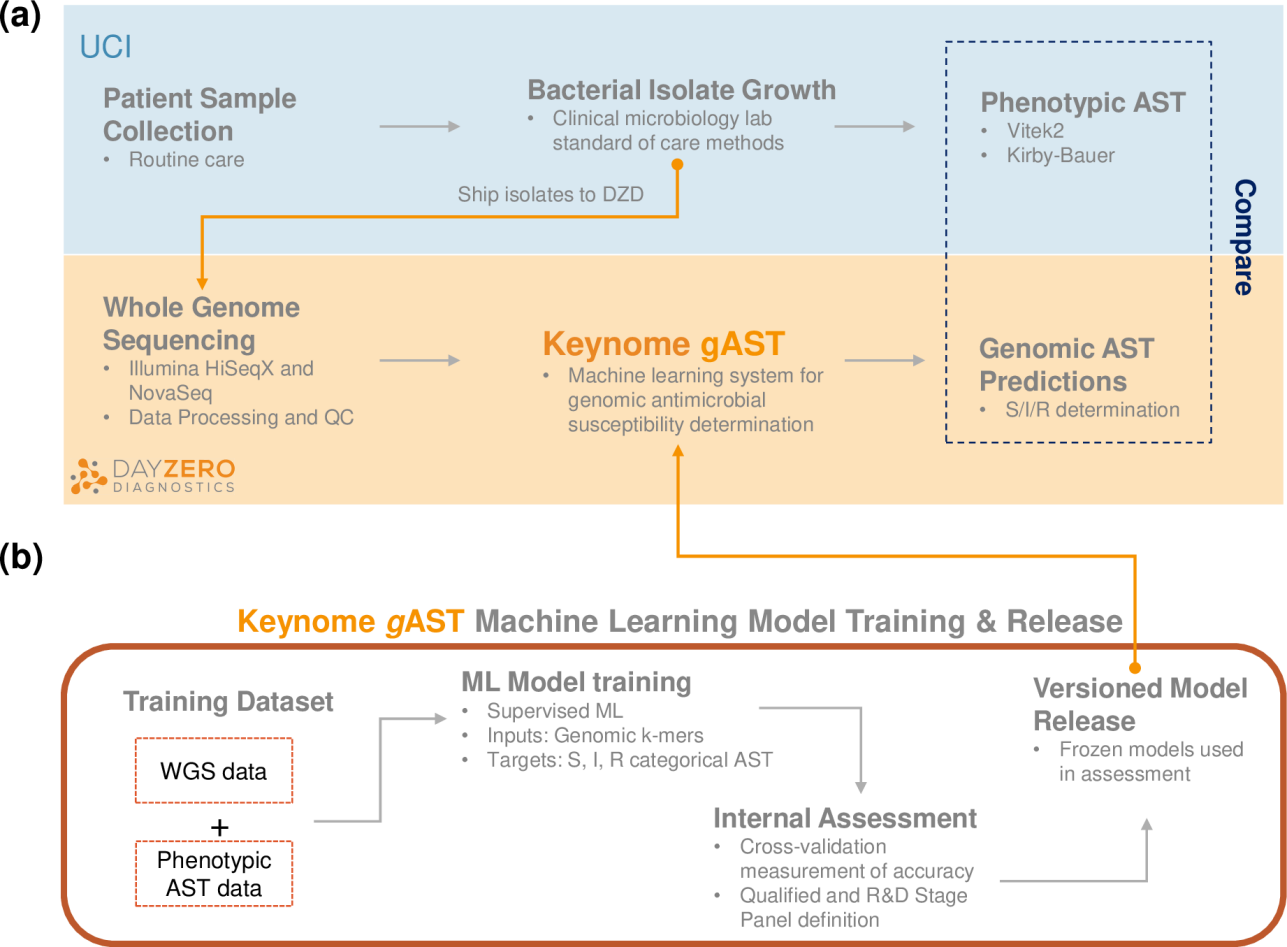
Experiment outline. (**a**) UCI collected bacterial isolates from routine care and shipped them to Day Zero Diagnostics. Isolates were sequenced and then processed with Keynome *g*AST, a ML system for genomic AST that predicts S/I/R phenotype for relevant drugs. Predicted ASTs were compared to phenotypic AST obtained in the clinical microbiology laboratory at UCI. (**b**) Keynome *g*AST underwent training prior to the assessment. After model training, internal accuracy assessment categorized each model as Qualified or R&D Stage. The models and Qualified/R&D Stage panels were frozen in a versioned release.

Of the isolates collected at the UCI Medical Center, 956 met the requirements for inclusion in the test data set for this study. WGS data from the isolates were processed by Keynome *g*AST, with phenotypic AST results available for 52 species-drug combinations from the Qualified panel and 45 species-drug combinations from the R&D Stage panel. Keynome *g*AST predicted 7,801 AST results across 18 distinct species and 23 distinct drugs (Fig. 1; Table 1; Table S1). Representation by species was strongly biased toward a small number of species, with the top three species*—E. coli*, *S. aureus*, and *K. pneumoniae*—comprising roughly two-thirds of the data set (65.7%), while many other species were only represented by a small number of samples; e.g., *S. agalactiae* (*n* = 1), *S. pneumoniae* (*n* = 2), and *S. lugdunensis* (*n* = 3).

**TABLE 1:** Test data set composition^*a*^

Species	# Isolates	# Antibiotics	# ASTs predicted
*Acinetobacter baumannii*	7	11	71
*Citrobacter freundii*	12	3	36
*Enterobacter cloacae*	17	5	85
*Enterococcus faecalis*	79	5	385
*Enterococcus faecium*	18	5	90
*Escherichia coli*	333	14	3,840
*Klebsiella michiganensis*	9	1	9
*Klebsiella pneumoniae*	104	12	1,126
*Klebsiella quasipneumoniae*	10	2	20
*Morganella morganii*	11	4	44
*Proteus mirabilis*	44	6	264
*Pseudomonas aeruginosa*	79	3	237
*Staphylococcus aureus*	191	7	1,336
*Staphylococcus epidermidis*	33	7	231
*Staphylococcus hominis*	3	3	9
*Staphylococcus lugdunensis*	3	3	9
*Streptococcus agalactiae*	1	3	3
*Streptococcus pneumoniae*	2	3	6

^*a*^ Numbers of isolates, antibiotics, and antimicrobial susceptibility tests in the final test data set are shown for each species.

In aggregate across the entire data set, the Keynome *g*AST Qualified panel displayed categorical agreement (CA) of 96.9% (Table 2). Major error (ME, phenotypic S predicted as R) and very major error (vME, phenotypic R predicted as S) rates were <2% with the majority of errors being minor errors (mE, any error with prediction or phenotype of I). Notably, these aggregate accuracy metrics would meet the FDA criteria (16) for phenotypic AST systems of CA > 90%, mE rate <3%, and vME rate <1.5%–7.5% (CA, mE rate: binomial test, *ɑ* = 0.05; vME rate: 95% confidence interval thresholds). The R&D Stage panel had expectedly lower aggregate accuracy, with CA of 88.5% and ME/vME rates < 5%, again with most errors being mEs, and would not meet the FDA criteria due to higher vME and mE rates.

**TABLE 2:** Keynome *g*AST accuracy^*a*^^,^^*b*^

Analysis level	Aggregate			Species-drug	
	Qualified panel	R&D stage panel		Qualified panel	R&D stage panel
**Sample sizes**			**Sample Sizes**		
# models	52	45	# models	24	9
# species | # drugs	15 | 17	17 | 19	# species | # drugs	6 | 11	5 | 5
# isolates	925	921	# isolates	N/A	N/A
# ASTs	5118	2683	# ASTs	N/A	N/A
# S | # I | # R	3576 | 98 | 1444	2187 | 123 | 373	# S | # I | # R	N/A	N/A
**Categorical Agreement**			**Categorical Agreement**		
Estimate (# Correct / Total)	96.9% (4957/5118)	88.5% (2374/2683)	Median	97.4%	86.7%
95% CI	96.3%–97.3%	87.2%–89.6%	IQR	95.3%–99.5%	85.6%–90.9%
**Very Major Error Rate**			**Very Major Error Rate**		
Estimate (# Errors / # Total)	1.4% (20/1444)	4.6% (17/373)	Median	0.0%	2.9%
95% CI	0.90%–2.1%	2.9%–7.2%	IQR	0.0%–2.8%	0.0%–7.7%
**Major Error Rate**			**Major Error Rate**		
Estimate (# Errors / # Total)	0.8% (28/3576)	1.0% (21/2187)	Median	0.4%	1.3%
95% CI	0.5%–1.1%	0.6%–1.5%	IQR	0.0%–0.8%	0.0%–3.2%
**Minor Error Rate**			**Minor Error Rate**		
Estimate (# Errors / # Total)	2.2% (113/5118)	10.1% (271/2683)	Median	1.0%	8.7%
95% CI	1.8%–2.7%	9.0%–1.3%	IQR	0.0%–3.1%	3.8%–11.5%

^*a*^ Keynome *g*AST performance was assessed at the aggregate level, across all samples and species-drug combinations; and at the individual species-drug combination level, where the analysis is restricted to data sets with at least 10 R and 10 S samples and the calculation shows the median across all data sets assessed. Accuracy metrics are shown for both the Qualified and R&D Stage panels. All confidence intervals are 95% binomial proportion confidence intervals. AST, antimicrobial susceptibility test; CI, confidence interval; IQR, interquartile range.

^*b*^ N/A, not applicable.

**Fig 2:**
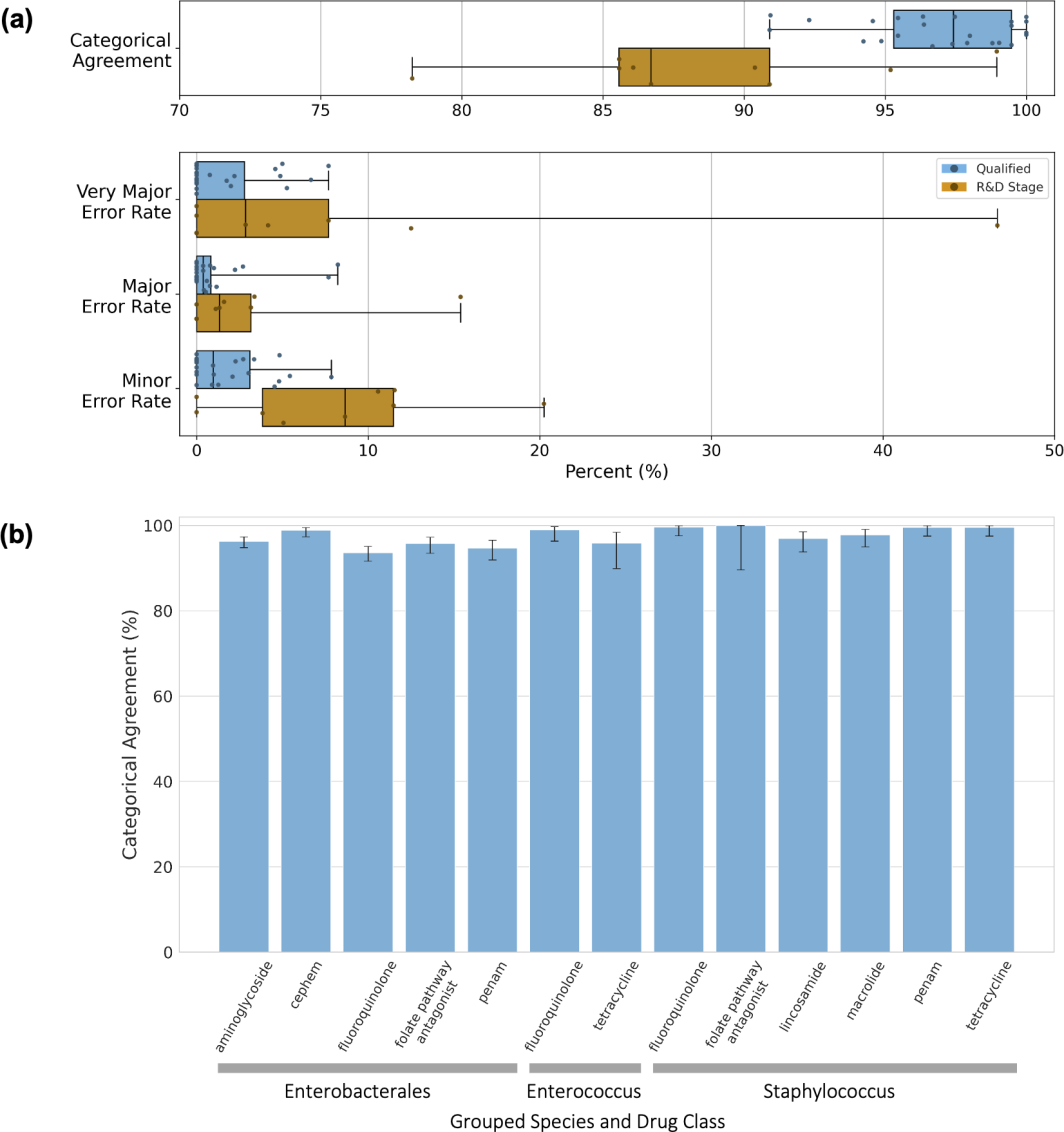
Keynome *g*AST performance. (**a**) Keynome *g*AST per model performance metrics across the 33 of 97 species-drugs with at least 10 R and 10 S samples in the test data set. Boxes represent interquartile range, the line within the box represents the median, and whiskers represent range of all data points. (**b**) Keynome *g*AST categorical agreement aggregated across Qualified models by species group and drug class with at least 10 R and 10 S samples. Error bars represent 95% binomial proportion confidence intervals.

We examined the performance of Keynome *g*AST on individual species-drug combinations with sufficient data set size for meaningful evaluation. On species-drug combinations with at least 10 R and 10 S samples, the Qualified panel had a median CA of 97.4% (IQR: 95.3%–99.5%), while the R&D Stage panel had a median CA 86.7% (IQR: 85.6%–90.9%) (Tables 2 and 3; Fig. 2a). Comparing performance at the species-drug level to the FDA criteria, a small number of combinations would pass (3 from the Qualified panel) or fail (5 from the Qualified panel, 10 from the R&D Stage panel), but the large majority (81.4%, 79/97) have insufficient data set sizes to make a conclusion with statistical significance (Table S2). Looking only at CA, where the sample sizes are largest due to including both R and S samples, all Qualified panel combinations that achieved statistical significance passed the >90% threshold (22 of 52 species-drugs); for the 45 R&D Stage combinations, 3 pass and 6 fail (with the remainder not reaching statistical significance).

**TABLE 3:** Keynome *g*AST performance by combination^*a*^

Panel	Species	Drug	Categorical agreement	Very major error rate	Major error rate	Minor error rate
Qualified Panel	*Enterococcus faecalis*	Tetracycline	97.5% (91.2%–99.3%)	2.0% (0.4%–10.5%)	0.0% (0.0%–11.7%)	1.3% (0.2%–6.8%)
	*Escherichia coli*	Ampicillin	94.6% (91.6%–96.5%)	0.0% (0.0%–1.8%)	0.0% (0.0%–3.2%)	5.4% (3.5%–8.4%)
		Ceftriaxone	98.8% (97.0%–99.5%)	0.0% (0.0%–3.7%)	0.4% (0.1%–2.4%)	0.9% (0.3%–2.6%)
		Ciprofloxacin	90.9% (87.4%–93.6%)	2.2% (0.8%–6.3%)	0.5% (0.1%–3.0%)	7.9% (5.4%–11.3%)
		Gentamicin	96.3% (93.7%–97.9%)	0.0% (0.0%–5.5%)	0.4% (0.1%–2.1%)	3.4% (1.9%–5.9%)
		Levofloxacin	94.9% (91.9%–96.8%)	0.8% (0.1%–4.2%)	0.0% (0.0%–2.3%)	4.8% (3.0%–7.7%)
		Tobramycin	96.4% (93.8%–97.9%)	4.9% (1.3%–16.1%)	0.4% (0.1%–2.1%)	2.7% (1.4%–5.1%)
		Trimethoprim/sulfamethoxazole	96.7% (94.1%–98.1%)	4.6% (2.0%–10.3%)	2.7% (1.2%–5.8%)	0.0% (0.0%–1.1%)
	*Klebsiella pneumoniae*	Ceftriaxone	99.0% (94.8%–99.8%)	0.0% (0.0%–16.1%)	0.0% (0.0%–4.4%)	1.0% (0.2%–5.2%)
		Gentamicin	98.0% (93.0%–99.4%)	7.7% (1.4%–33.3%)	0.0% (0.0%–4.2%)	1.0% (0.2%–5.4%)
		Tobramycin	94.2% (88.0%–97.3%)	0.0% (0.0%–22.8%)	1.2% (0.2%–6.3%)	4.8% (2.1%–10.8%)
		Trimethoprim/sulfamethoxazole	92.3% (85.6%–96.1%)	5.3% (0.9%–24.6%)	8.2% (4.0%–16.0%)	0.0% (0.0%–3.6%)
	*Proteus mirabilis*	Ampicillin	95.5% (84.9%–98.7%)	6.7% (1.2%–29.8%)	0.0% (0.0%–11.7%)	2.3% (0.4%–11.8%)
		Ciprofloxacin	100.0% (92.0%–100.0%)	0.0% (0.0%–16.1%)	0.0% (0.0%–13.8%)	0.0% (0.0%–8.0%)
		Levofloxacin	95.5% (84.9%–98.7%)	0.0% (0.0%–17.6%)	0.0% (0.0%–14.3%)	4.5% (1.3%–15.1%)
	*Staphylococcus aureus*	Clindamycin	97.9% (94.7%–99.2%)	1.8% (0.3%–9.3%)	2.2% (0.8%–6.4%)	0.0% (0.0%–2.0%)
		Erythromycin	97.4% (94.0%–98.9%)	0.0% (0.0%–4.1%)	1.0% (0.2%–5.5%)	2.1% (0.8%–5.3%)
		Levofloxacin	99.5% (97.1%–99.9%)	0.0% (0.0%–5.9%)	0.8% (0.1%–4.3%)	0.0% (0.0%–2.0%)
		Oxacillin	99.5% (97.1%–99.9%)	0.0% (0.0%–5.7%)	0.8% (0.1%–4.3%)	0.0% (0.0%–2.0%)
		Tetracycline	99.5% (97.1%–99.9%)	0.0% (0.0%–13.8%)	0.6% (0.1%–3.3%)	0.0% (0.0%–2.0%)
	*Staphylococcus epidermidis*	Clindamycin	90.9% (76.4%–96.9%)	5.0% (0.9%–23.6%)	7.7% (1.4%–33.3%)	3.0% (0.5%–15.3%)
		Levofloxacin	100.0% (89.6%–100.0%)	0.0% (0.0%–20.4%)	0.0% (0.0%–17.6%)	0.0% (0.0%–10.4%)
		Oxacillin	100.0% (89.6%–100.0%)	0.0% (0.0%–14.3%)	0.0% (0.0%–27.8%)	0.0% (0.0%–10.4%)
		Trimethoprim/sulfamethoxazole	100.0% (89.6%–100.0%)	0.0% (0.0%–22.8%)	0.0% (0.0%–16.1%)	0.0% (0.0%–10.4%)
R&D Stage Panel	*Escherichia coli*	Ampicillin/sulbactam	78.2% (73.5%–82.4%)	0.0% (0.0%–3.3%)	3.2% (1.4%–7.2%)	20.2% (16.3%–24.9%)
		Cefepime	86.7% (82.6%–89.9%)	2.9% (0.8%–9.8%)	1.6% (0.6%–4.0%)	11.5% (8.5%–15.4%)
	*Klebsiella pneumoniae*	Ampicillin/sulbactam	85.6% (77.6%–91.1%)	12.5% (4.3%–31.0%)	1.3% (0.2%–7.2%)	10.6% (6.0%–18.0%)
		Cefepime	85.6% (77.6%–91.1%)	0.0% (0.0%–21.5%)	3.4% (1.2%–9.4%)	11.5% (6.7%–19.1%)
		Ciprofloxacin	95.2% (89.2%–97.9%)	4.2% (0.7%–20.2%)	0.0% (0.0%–4.8%)	3.8% (1.5%–9.5%)
		Levofloxacin	90.4% (83.2%–94.7%)	7.7% (1.4%–33.3%)	0.0% (0.0%–4.9%)	8.7% (4.6%–15.6%)
	*Proteus mirabilis*	Trimethoprim/sulfamethoxazole	90.9% (78.8%–96.4%)	0.0% (0.0%–17.6%)	15.4% (6.2%–33.5%)	0.0% (0.0%–8.0%)
	*Pseudomonas aeruginosa*	Ciprofloxacin	86.1% (76.8%–92.0%)	46.7% (24.8%–69.9%)	0.0% (0.0%–6.0%)	5.1% (2.0%–12.3%)
	*Staphylococcus aureus*	Trimethoprim/sulfamethoxazole	99.0% (96.3%–99.7%)	0.0% (0.0%–24.2%)	1.1% (0.3%–4.0%)	0.0% (0.0%–2.0%)

^*a*^ Keynome *g*AST performance relative to ground truth AST phenotype and binomial proportion confidence interval (CI), for Qualified and R&D Stage panels restricted to data sets with at least 10 R and 10 S samples in the final test data set.

To strike a balance between sample size and the level of similarity among aggregated samples, we also assessed performance at a mid-level of aggregation by grouping both species and drugs into clinically relevant classes (Fig. 2b; Table S3). At this level of analysis over half (13/23) of combinations on the Qualified panel met the FDA > 90% CA criterion and none failed, with the rest still having sample sizes too small to make a statistically significant determination.

**Fig 3:**
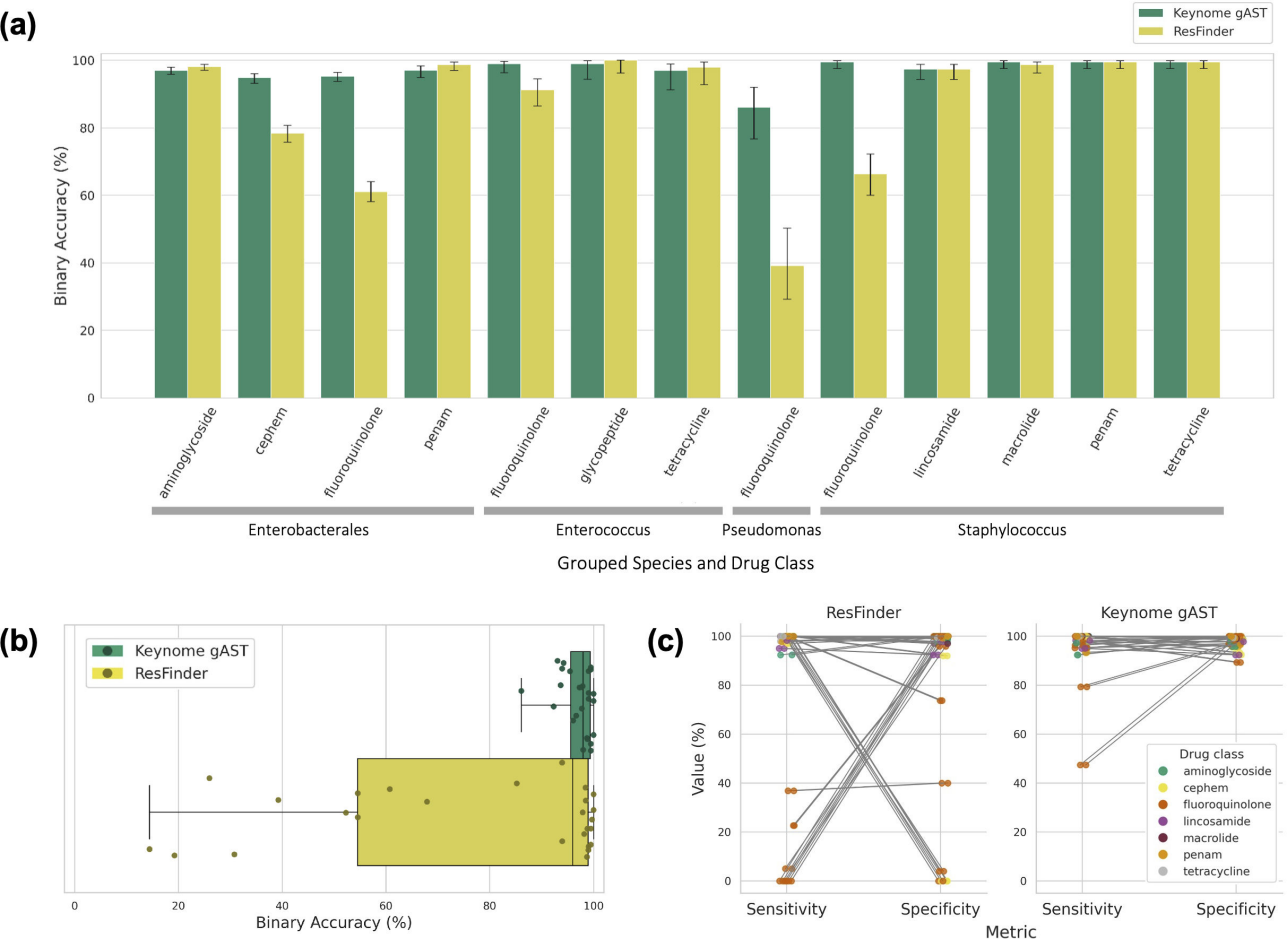
Keynome *g*AST vs ResFinder performance. (**a**) Keynome *g*AST and ResFinder binary accuracy aggregated across models within the same species group and drug class with at least 10 R and 10 S samples in the test data set. Error bars represent 95% binomial proportion confidence intervals. (**b**) Per model S/NS binary accuracy of Keynome *g*AST and ResFinder across the 26 of 83 species-drugs with at least 10 R and 10 S samples in the test data set and predictions from both methods. Boxes represent interquartile range, the line within the box represents the median, and whiskers represent range of all data points. (**c**) Per model sensitivity and specificity trade-off of Keynome *g*AST and ResFinder across the same set of species-drugs shown in (**b**).

We compared the prediction accuracy of Keynome *g*AST with that of a resistance marker approach, ResFinder. We restricted the analysis to the 83 of 97 species-drug combinations where ResFinder has annotations for relevant markers, comprising 947 isolates with 6,553 ASTs (Table S4). Since ResFinder does not distinguish intermediate from resistance phenotypes, we grouped intermediate and resistant AST phenotypes and predictions into a non-susceptible (NS) category. On this binary S/NS task, Keynome gAST achieved 96.0% aggregate accuracy, significantly outperforming ResFinder’s 83.5% (a 12.5 percentage point difference; *P* < 0.001, McNemar’s test). This performance gap was also evident across major drug and species classes (Fig. 3a; Table S5), including significant differences in: Enterobacterales/carbapenem (*P* < 0.001), cephem (*P* < 0.001), and fluoroquinolone (*P* < 0.001); Enterococcus/fluoroquinolone (*P* = 0.003); Pseudomonas/fluoroquinolone (*P* < 0.001); Staphylococcus/fluoroquinolone (*P* < 0.001).

Evaluating the individual species-drug combinations (for those with at least 10 S and R samples), the median binary accuracy of Keynome gAST (98.0%) was comparable to ResFinder (96.1%). However, ResFinder’s performance was far more variable (IQR: 50.1%–99.0%) than Keynome *g*AST’s (IQR: 95.7%–99.5%), with many combinations performing at or near chance (50% accuracy) (Fig. 3b). This variability stemmed from a stark trade-off in ResFinder between sensitivity and specificity, with one metric high while the other is low, a phenomenon Keynome *g*AST largely avoided (Fig. 3c).

This trade-off between sensitivity and specificity is a consequence of ResFinder’s database-driven method. Low sensitivity arose from an incomplete marker database, particularly for fluoroquinolones due to a systematic lack of point mutations annotated as relevant to levofloxacin. Conversely, low specificity resulted from the inclusion in the database of markers with low positive predictive value (PPV), whose presence did not reliably confer a non-susceptible phenotype.

**Fig 4:**
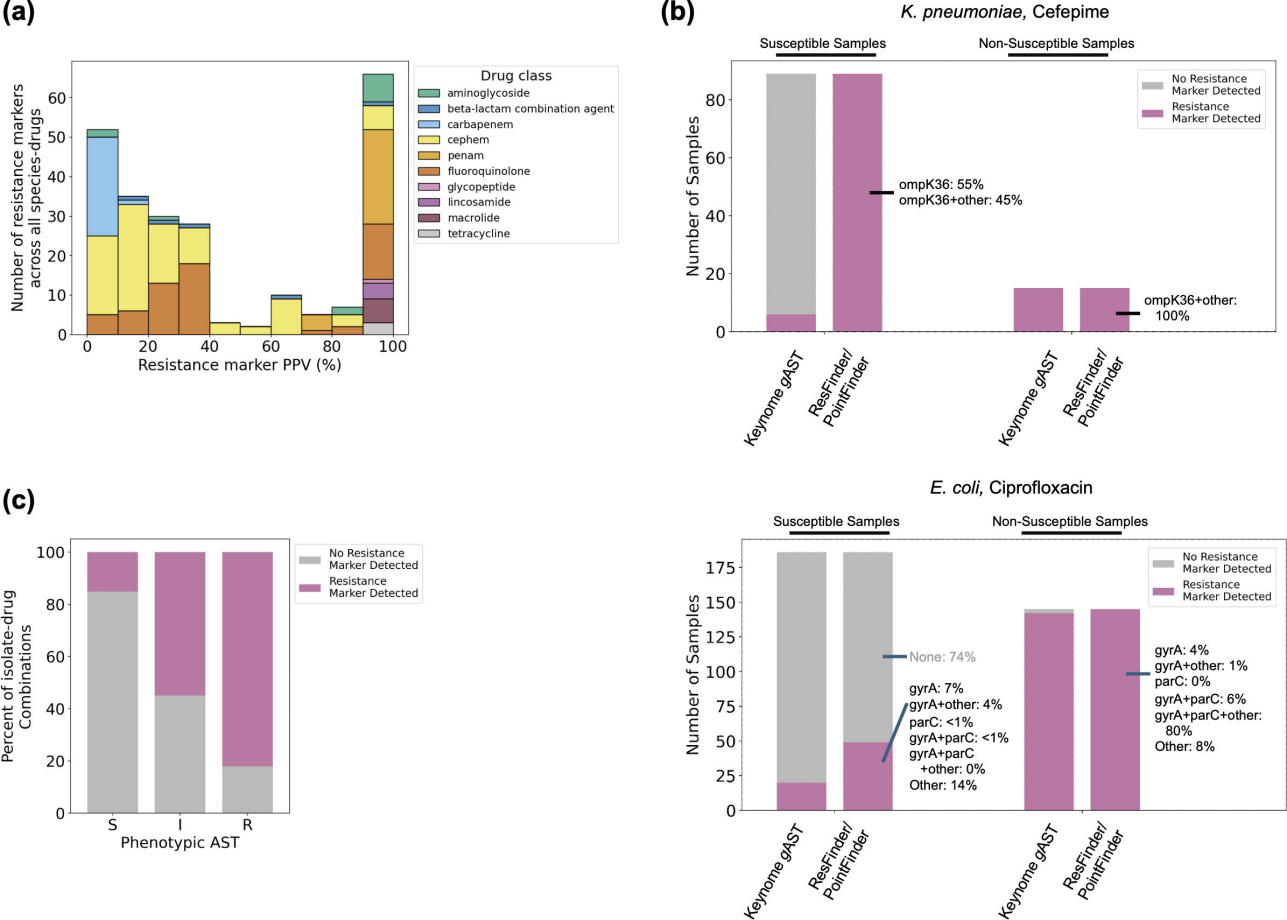
Resistance marker limitations analysis. (**a**) Histogram of positive predictive value (PPV) of individual ResFinder resistance markers. PPV was computed for each marker in each relevant species-drug, restricted to resistance marker combinations found in at least 10 samples in the species-drug data set. (**b**) Upper: Keynome *g*AST and ResFinder predictions on the *K. pneumoniae*—cefepime data set. ResFinder exhibited 0% specificity due to ubiquitous presence of *ompK* porin gene mutations in susceptible samples. Lower: Keynome *g*AST and ResFinder predictions on the *E. coli*—ciprofloxacin data set. ResFinder exhibited a specificity of 72.2% due to the high number of annotated mutations in DNA gyrase and topoisomerase genes *gyrA* and *parE* in susceptible samples. (**c**) The presence of resistance markers as determined by ResFinder in phenotypically susceptible (S), intermediate (I), and resistant (R) samples; percent of all isolate-drug combinations with or without a marker is shown for each phenotypic group.

Many markers had a PPV between 0% and 50% (Fig. 4a; Table S6), driving down specificity. Examples of such low-PPV markers include particular mutations in the *K. pneumoniae* outer membrane porins *ompK36* and *ompK37* linked by ResFinder to beta-lactam resistance (Fig. 4b, upper panel); and a variety of markers linked to ciprofloxacin resistance in Gram-negative species, such as certain mutations in DNA gyrase and topoisomerase genes (*gyrA*, *parE*) (17) (Fig. 4b, lower panel), mutations in the efflux pump repressor gene *AcrR* (18, 19), efflux pump genes (*OqxA/B*) (20), and the *Pseudomonas* ciprofloxacin-modifying enzyme *crpP* (21, 22). Although many of these markers are known to be insufficient for causing resistance on their own, ResFinder’s rigid approach predicts non-susceptibility based on the presence of any single marker. While using marker combinations could theoretically improve accuracy, this approach is practically challenging due to the vast number of combinations and their variable predictive values (Fig. S1; Table S7).

The inflexibility of this marker-based approach is highlighted by the discordance between phenotypes and marker presence. ResFinder detected a resistance marker in only 82.0% of resistant and 55.0% of intermediate isolates, while detecting markers in 15.1% of susceptible isolates (Fig. 4c). Keynome *g*AST performance was notably better when the phenotype was concordant with resistance marker presence or absence but still had reliable performance in the discordant cases (Table 4). For instance, its vME rate was 1.1% on concordant samples and 5.2% on discordant samples. By its nature, ResFinder is unable to resolve these discordant cases and cannot make intermediate predictions.

**TABLE 4:** Keynome *g*AST performance stratified by the presence or absence of resistance markers^*a*^^,^^*b*^

AST phenotype	Resistance marker(s) present	Resistance marker concordance with phenotype	# Predictions	Keynome*g*AST categorical agreement	Keynome*g*AST major error rate	Keynome*g*AST very major error rate
R	True	Concordant	1,219	93.4% (93.4%–93.5%)	N/A	1.1% (1.1%–1.2%)
	False	Discordant	267	92.5% (92.4%–92.6%)	N/A	5.2% (5.2%–5.3%)
I	True	Concordant	83	81.9% (81.7%–82.2%)	N/A	N/A
	False	Discordant	68	58.8% (58.4%–59.2%)	N/A	N/A
S	True	Discordant	744	89.7% (89.6%–89.7%)	2.69% (2.65%–2.73%)	N/A
	False	Concordant	4,172	96.98% (96.96%–97.00%)	0.072% (0.069%–0.075%)	N/A

^*a*^ Keynome *g*AST performance on samples in the test data set, stratified by AST phenotype and presence/absence of resistance marker as identified by ResFinder.

^*b*^ N/A, not applicable.

## DISCUSSION

In this study, we assessed the performance of Keynome *g*AST, a machine learning genomic AST system, on a clinically representative collection of over 900 bacterial isolates with over 7,000 ground truth AST results from a single US medical center. Across the entire data set, the Keynome *g*AST Qualified panel achieved >96% categorical agreement (CA), with very major error (vME; phenotypic R predicted as S) rate 1.4%, and major error (ME; phenotypic S predicted as R) rate 0.8% (Table 2; Fig. 2). Across individual species-drug combinations with suitable representation in the data set (≥10 R and ≥10 S samples), performance of this panel was consistently high, with a median categorical agreement of 97.4%. The Keynome *g*AST R&D Stage panel demonstrated expectedly lower performance, driven by increases in both very major error and minor error rates.

We also compared Keynome *g*AST to ResFinder, a baseline resistance marker approach, using a susceptible/non-susceptible binary prediction task. Across the full data set, Keynome *g*AST performed significantly better than ResFinder, with an overall accuracy of 96.0% compared to 83.5%. When investigated at the level of individual species-drug combinations, this difference was attributed to ResFinder frequently making poor sensitivity/specificity trade-offs (Fig. 3c). This was driven by the presence of resistance markers that were insufficient to imply resistance (low specificity) or a lack of relevant markers annotated for a particular combination (low sensitivity). Across the full data set, ResFinder detected a resistance marker in 15.1% of phenotypically susceptible samples and failed to detect a resistance marker in 18.0% of phenotypically resistant samples (Fig. 4c). In contrast, Keynome *g*AST still achieved good performance on both phenotypically susceptible samples with a marker (89.7% CA, 2.7% ME rate) and phenotypically resistant samples without a marker (92.5% CA, 5.2% vME rate; Table 4).

While ResFinder is an invaluable tool for understanding determinants of resistance in a research setting, its predictive logic—classifying an isolate as resistant if any single marker is detected—is too simplistic for many cases of more complex resistance mechanisms. A more robust strategy for genomic AST involves assessing combinations of resistance markers (Table S7). However, such an approach is challenging due to the combinatorial explosion of possible cases, and hand-designing rule sets requires deep expertise resulting in solutions siloed by species (23–27).

Alternatively, machine learning (ML) offers a flexible, data-driven approach for genomic AST prediction capable of learning complex resistance mechanisms by incorporating interactions between genetic loci. Furthermore, an ML approach such as Keynome *g*AST need not be constrained to relying on known resistance markers, which are limited by previous scientific characterization and database curation. Instead, it can flexibly characterize the entire genome in order to discover the most predictive features *de novo* for each species-drug combination. ML genomic AST solutions have been investigated (28–33), but unfortunately, no comprehensive models are readily available at this time. For these reasons, users frequently default to tools like ResFinder for genomic AST predictions despite their limitations. It is also important to note that ResFinder only explicitly supports a subset of the species in the data set (78% of isolates), but a similar analysis restricted to these species-drug pairs yielded similar qualitative conclusions (see Fig. S2 and S3; Methods).

Other ML methods for genomic AST have been developed that vary in terms of featurization of the WGS data, ML model, training algorithm, and prediction targets and have been thoroughly reviewed elsewhere (8, 34). Most ML genomic AST methods predict categorical AST, though predicting MIC is an area of active investigation with some promising results on limited species (33, 35–37). There are other categorical genomic AST prediction methods similar to Keynome *g*AST that also employ *k*-mers as input features for boosted regression and classification trees (9–13) though Keynome *g*AST differs from these approaches in two key ways. First, it predicts intermediate and resistant phenotypes separately, as opposed to a combined non-susceptible phenotype. Second, it was trained on an expansive and highly curated data set which contained 418,218 AST measurements from 42,976 unique isolates, spanning 265 species-drug combinations across 24 distinct species. While many studies use training data based exclusively from public aggregators such as BV-BRC or NDARO (33, 38) and/or focus on only a single pathogen species (39, 40), almost 2/3 of the ASTs in the Keynome *g*AST training data set were generated through sourcing from clinical collaborators and biobanks, targeting breadth and diversity of clinically important pathogens, with all samples undergoing extensive quality control and data harmonization.

In this assessment of Keynome *g*AST, the composition of the test data set is both a strength and a weakness. As isolates were broadly collected across departments within a single clinical site over a relatively short timeframe, the collection is naturally representative of what would be encountered in this clinically relevant setting—in terms of species, drugs tested, and AST phenotypes observed—and aggregate performance results can be interpreted in this light. However, this also means that the species and phenotypes represented are strongly biased toward those most often encountered in this particular setting and aggregate performance measures are correspondingly biased toward those combinations. In spite of this, we found a high level of within-species heterogeneity with a diversity of sequence types represented for the most common species (Fig. S4; Table S8). Other limitations of the data set include a general lack of challenging genotypes and/or phenotypes (only 26.1% overall non-susceptible) and the reliance of a non-gold-standard reference method (Vitek 2) for ground truth phenotypic AST (41, 42).

An important aspect when considering the clinical utility of genomic AST systems such as Keynome *g*AST is the ability to be integrated into systems that operate directly from clinical samples. While this study applied genomic AST to sequenced isolated strains, it would be more impactful to integrate the method with direct-from-sample pathogen DNA enrichment and sequencing, thereby enabling rapid predictive AST to guide therapeutic selection in hours rather than days by bypassing the need for traditional culturing and subculturing. Recent work, such as references 43, 44, and 45, have begun to demonstrate the feasibility of applying Keynome *g*AST along with other bioinformatics pipelines directly to sequenced bloodstream and respiratory samples.

One future direction of this work is improving the breadth and accuracy of Keynome *g*AST to expand the representation of clinically significant species-drug combinations on the Qualified panel. Particularly notable omissions include vancomycin for many gram positive species and beta-lactam/inhibitor combination therapies for gram negative species. Some of the missing species-drug combinations currently lack sufficiently large training data sets, particularly for rare resistant phenotypes (e.g., *S. aureus*/vancomycin), suggesting that improved performance may result from data sourcing. Other cases, such as many of the beta-lactam/inhibitor combination therapies, currently do not meet internal testing thresholds to qualify for the Keynome *g*AST panels. These combinations have proven challenging even with larger data sets, indicating that the method may require further innovations in the machine learning models or in additional genomic features such as gene copy number (46).

In summary, this study demonstrates the feasibility of a genomic AST ML system to provide high accuracy and extensive AST profiles on a wide set of species and drugs representative of those encountered in a US clinical microbiology lab.

## Data Availability

The sequencing data of the 956 bacterial isolates and corresponding AST data collected for this study can be accessed on the NCBI SRA database via BioProject accession number PRJNA1271593. All patient identifying information has been removed.

## SUPPLEMENTAL MATERIAL

Supplemental materials spectrum.00564-25-s0001.docx Supplemental methods, legend for Tables S1 to S8, and Figures S1 to S4.

Supplemental tables spectrum.00564-25-s0002.xlsx Tables S1 to S8.

ASM does not own the copyrights to Supplemental Material that may be linked to, or accessed through, an article. The authors have granted ASM a non-exclusive, world-wide license to publish the Supplemental Material files. Please contact the corresponding author directly for reuse.
