# Supplementary material for: Evaluation of a machine learning system for genomic antimicrobial susceptibility determination on a clinically representative test set
Source: Microbiol Spectr. 2026 Jan 27;14(3):e00564-25. doi: 10.1128/spectrum.00564-25 (PMC12955427; doi:10.1128/spectrum.00564-25)
Supplement: Supplemental materials — Supplemental methods, legend for Tables S1 to S8, and Figures S1 to S4. [file spectrum.00564-25-s0001.docx]

## Supplementary Materials

### Supplementary Methods:

#### Quality Control for Test Dataset Inclusion

Isolates from the test dataset were excluded based on the following criteria:

1. No AST results
2. Species identity, contamination, and completeness
   1. The genotypically determined species did not match that determined phenotypically at the genus level. In the cases of discrepant identities within the same genus, the genotypic determination was taken to be correct
   2. <80% trimmed reads consistent with the determined species
   3. De novo assembly with length or GC content outside the expected taxon-specific range based on a curated set of reference genomes.
3. Sequencing quality
   1. Low sequencing depth: <15x estimated depth based on average species-specific genome length
   2. Abnormally high/low number of 20-mers: >125% or <90% of the average species-specific genome length
4. Assembly quality
   1. Fragmented assembly: N_50_ <30kb; average contig length <5kb; or >30% of contigs less than or equal to 1kb
   2. Low depth of coverage: average depth on mapping back to de novo assembly <30x
   3. Low breadth of coverage: 5x coverage < 90%
   4. Unaligned reads: reads aligning to de novo assembly < 90%
5. Genomic Relatedness: Any isolates that were with closely related genomes to any samples in the Keynome *g*AST training datasets (based on the same k-mer based similarity measure used for the same purpose in the training data; see below).

The remaining isolates all had at least one (though frequently many) associated phenotypic AST results. These AST results were further subset for different analyses:

1. All AST results that did not match at least one Qualified or R&D Keynome *g*AST species-drug combination were excluded from further analysis
2. Further ASTs were excluded that did not have a quantitative value (MIC or zone diameter), and only an interpretive category (S/I/R), if there was ambiguity in which set of breakpoints were used to make the interpretation due to the existence of multiple breakpoint sets for different indications (e.g. urinary tract infection versus meningitis) or recently breakpoint changes in the CLSI standards.

These were the ASTs used to assess overall Keynome *g*AST performance. To compare Keynome *g*AST performance to that of ResFinder:

1. Further ASTs were excluded for species-drug combinations for which ResFinder had no annotations for linking any resistance marker to the species (or higher order taxa) or the drug (or higher order drug class) and thus could never make a positive identification.

#### Clonality Assessment

##### Similarity Score

To quantify the degree of similarity between the genomes of two isolates in a scalable fashion, the overlap (or Szymkiewicz-Simpson) coefficient between the canonical 20-mer sets of the genomes was employed, i.e.

$$similarity=\frac{\left| S_{1}\cap S_{2} \right|}{min\left( \left| S_{1} \right|,\left| S_{s} \right| \right)},$$

where S_1_ and S_2_ are the canonical k-mer sets of the two genomes, i.e. the lexicographically smaller of each k-mer present and its reverse complement, to avoid ambiguity between sense and antisense strand sequences. This similarity score between 0 and 1 represents the fraction shared k-mers relative to the size of the smaller genome. Using the smaller genome in the denominator ensures that genomes that mainly differ by the acquisition of a mobile element (e.g. a plasmid or phage) but are otherwise identical will still be characterized as highly similar. Genomes with a similarity score >0.99975 (i.e. <0.025% shared 20-mers, assuming identical genome sizes) were considered clonal for the purposes of both model training and test dataset curation.

#### Machine Learning Models

##### Training Dataset

The training data is composed of a collection of short read sequencing data derived from single-colony isolates, each of which is paired with phenotypic species identification and laboratory-derived AST results for one or more antibiotics. Clinical bacterial isolates were sourced from clinical sites, biobanks and private collections over the course of 8 years. Phenotypic AST typically was performed by the isolate collection source, and Illumina WGS of the isolates was performed by Day Zero Diagnostics. Previously sequenced isolates were also sourced from publicly available repositories such as PATRIC [(now BV-BRC, 1)](https://www.zotero.org/google-docs/?4IVt9B) and NDARO [(2)](https://www.zotero.org/google-docs/?IKEAzj). When possible, DZD reinterpreted AST determination of Susceptible (S), Intermediate (I), or Resistant (R) from the raw measurement (MIC for broth microdilution or zone diameter for Kirby-Bauer) using CLSI M100 breakpoints (32nd edition). To identify redundant data within datasets due to clonality, we used a k-mer based method to assess the genomic similarity between isolates within a species-drug dataset and remove closely-related isolates (see above). The final aggregated training dataset that was used at the time of this study consisted of 42,976 unique isolate genomes and 418,218 phenotypic AST results. There were 265 individual datasets, one for each species-drug combination for which a model was trained, totaling 49 distinct drugs across 24 distinct species covering both Gram positive and negative species. No samples from the UCI test dataset described above were included in any of the training datasets.

##### Model Training

The Keynome *g*AST ML models used in this study employ a canonical k-mer representation of the genome derived from the WGS reads that specifies which nucleotide subsequences of a fixed integer length *k* (here, k=20) are present or absent from the given genome. K-mers with identical representation across the training dataset are grouped together to form a single feature. For each species-drug combination, a 2-by-2 chi-squared contingency analysis is performed between k-mer feature presence/absence and Susceptible / Not Susceptible AST phenotype for each k-mer feature to select up to the top 2 million features for use training. The ML models employ gradient-boosted classification and regression tree (CART) models to predict S/I/R AST result targets. These targets are encoded as {S: 0.0, I: 0.5, R: 1.0} or {S:0.0, R: 1.0} depending on whether or not a species-drug has an Intermediate phenotype defined; this enables the use of a standard mean-squared error training objective. Models are fit using the XGBoost [(3)](https://www.zotero.org/google-docs/?9RtXcV) software package. Numeric model predictions are interpreted via breakpoints at ⅓ and ⅔ (species-drugs with an Intermediate phenotype) or ½ (species-drugs without an Intermediate phenotype) to produce a final categorical prediction.

##### Internal Model Evaluation

Prior to model release to production systems, five-fold cross-validation was used to provide an internal assessment of each model’s expected performance based on aggregate metrics across all test folds, including error/agreement rates and their associated 95% binomial proportion confidence intervals (Wilson score interval). A model was included on the “Qualified” panel if the categorical agreement point estimate / confidence interval lower bound were >88%/76%; and the very major and major error rates point estimates / confidence interval upper bounds were <6%/12%. A model was included on the “R&D Stage” panel if the categorical agreement point estimate / confidence interval upper bound was >76%/52% and the very major and major error rate point estimates / confidence interval upper bounds were <12%/24%. Models which did not reach this level of accuracy in cross-validation testing were not considered.

###

### Supplementary Tables:

#### Table S1:

Keynome *g*AST models assessed, with panel assignments and training dataset sizes in the Qualified and R&D Stage panels, for each species

#### Table S2:

Keynome *g*AST performance per species and drug for the Qualified and R&D Stage panels, comparing each against FDA benchmarks

#### Table S3:

Keynome *g*AST performance per species group and drug class combination for the Qualified and R&D Stage panels

#### Table S4:

Keynome *g*AST vs ResFinder binary (S/NS) performance per species and drug for the Qualified and R&D Stage panels

#### Table S5:

Keynome *g*AST vs ResFinder binary (S/NS) performance per species group and drug class for the Qualified and R&D Stage panels

#### Table S6:

Positive predictive value of individual resistance markers in relevant species-drug combinations

#### Table S7:

Positive predictive value of resistance marker combinations in relevant species-drug combinations

#### Table S8:

Sequence type counts per species

### Supplementary Figures

#### Figure S1:

Histogram of positive predictive value (PPV) of ResFinder resistance markers combinations; PPV computed for each marker combination in each relevant species-drug, restricted to resistance marker combinations found in at least 10 samples in the species-drug dataset.


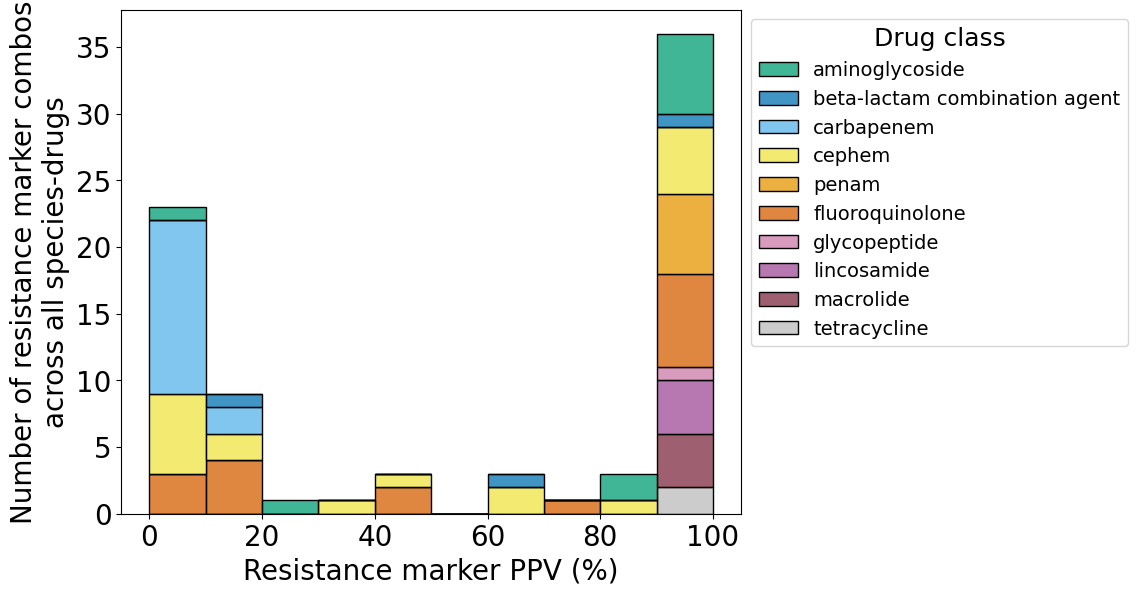


####

#### Figure S2: Keynome *g*AST vs ResFinder performance on PointFinder-supported taxa

This figure includes panels similar to those found in Figure 3, but shows results from an analysis restricted to taxa where PointFinder mutation databases are available (*E. faecalis*, *E. faecium, E. coli*, *Klebsiella*, and *S. aureus*) (Methods), which covers 78% of the isolates analyzed in the main text figures.

**(a)** Keynome *g*AST and ResFinder binary accuracy aggregated across models within the same species group and drug class. Error bars represent 95% binomial proportion confidence intervals.

**(b)** Per model S/NS binary accuracy of Keynome *g*AST and ResFinder across the 19 of 39 species-drugs with at least 10 R and 10 S samples in the test dataset and predictions from both methods. Boxes represent interquartile range and whiskers represent range of all data points.

**(c)** Per model sensitivity and specificity trade-off of Keynome *g*AST and ResFinder across the same set of species-drugs shown in **(b)**.


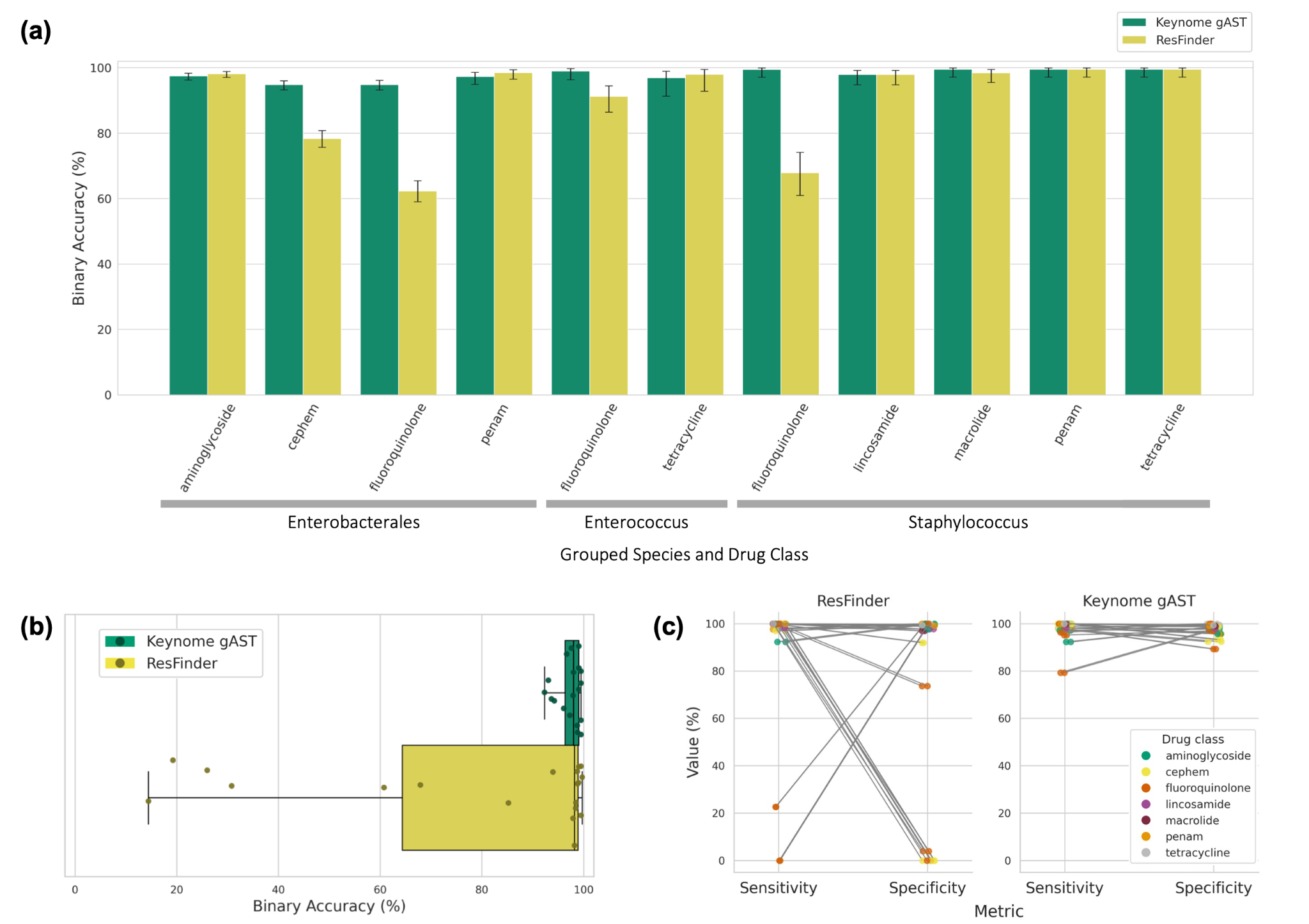


####

#### Figure S3

This figure is similar to Figure 4a, but shows results from an analysis restricted to taxa where PointFinder mutation databases are available (*E. faecalis, E. faecium, E. coli, Klebsiella,* and *S. aureus*) (Methods), which covers 78% of the isolates analyzed in the main text figures. The presence of resistance markers as determined by ResFinder in phenotypically Susceptible (S), Intermediate (I), and Resistant (R) samples; percent of all isolate-drug combinations with or without a marker is shown for each phenotypic group.


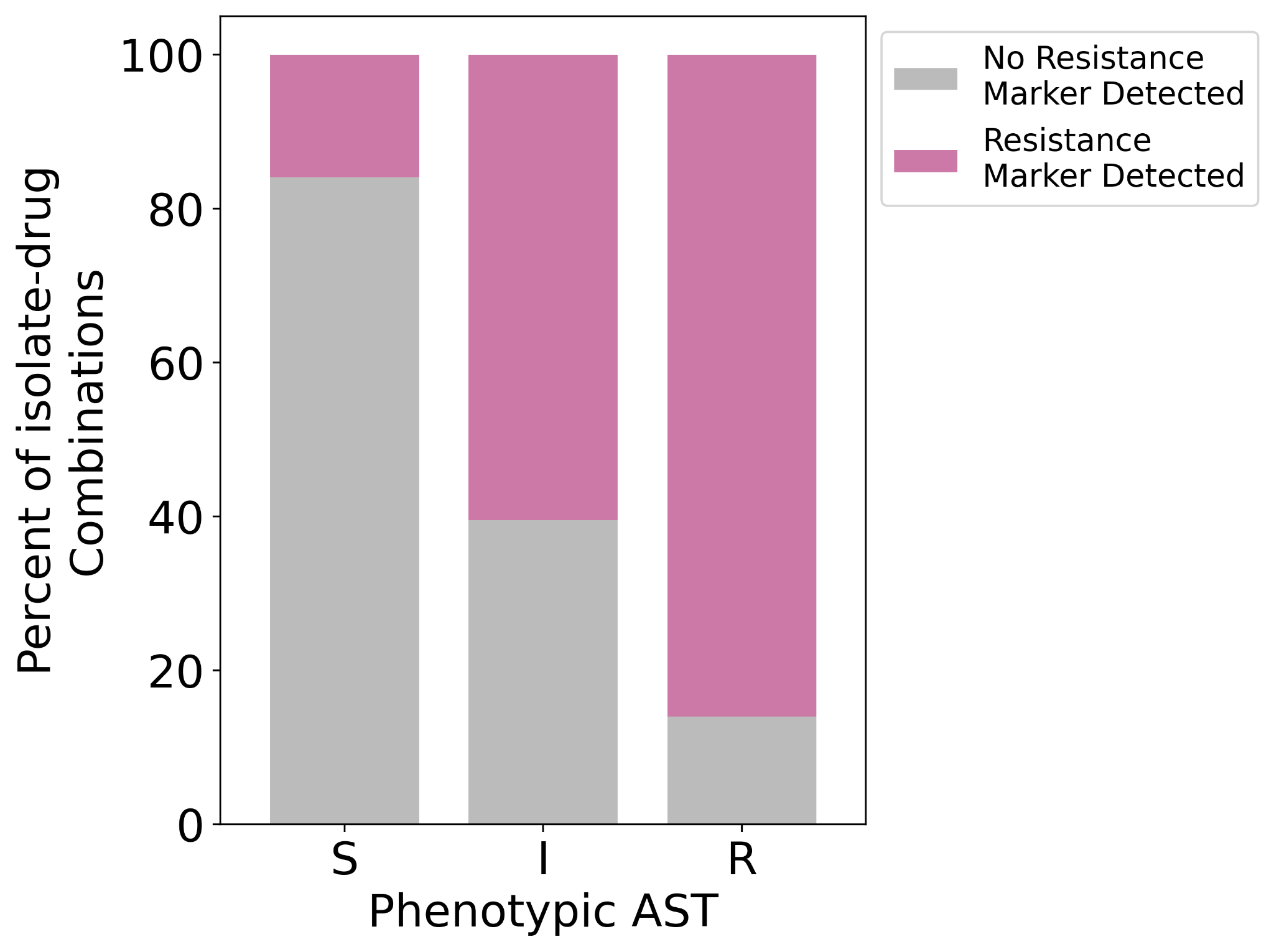


####

#### Figure S4: Sequence Types

Sequence type (ST) composition for the two most highly represented gram positive (*S. aureus* and *E. faecium*) and gram negative (*E. coli* and *K. pneumoniae*) species in the test dataset. The 20 largest ST’s are represented explicitly and the remainder are grouped into “Other”.


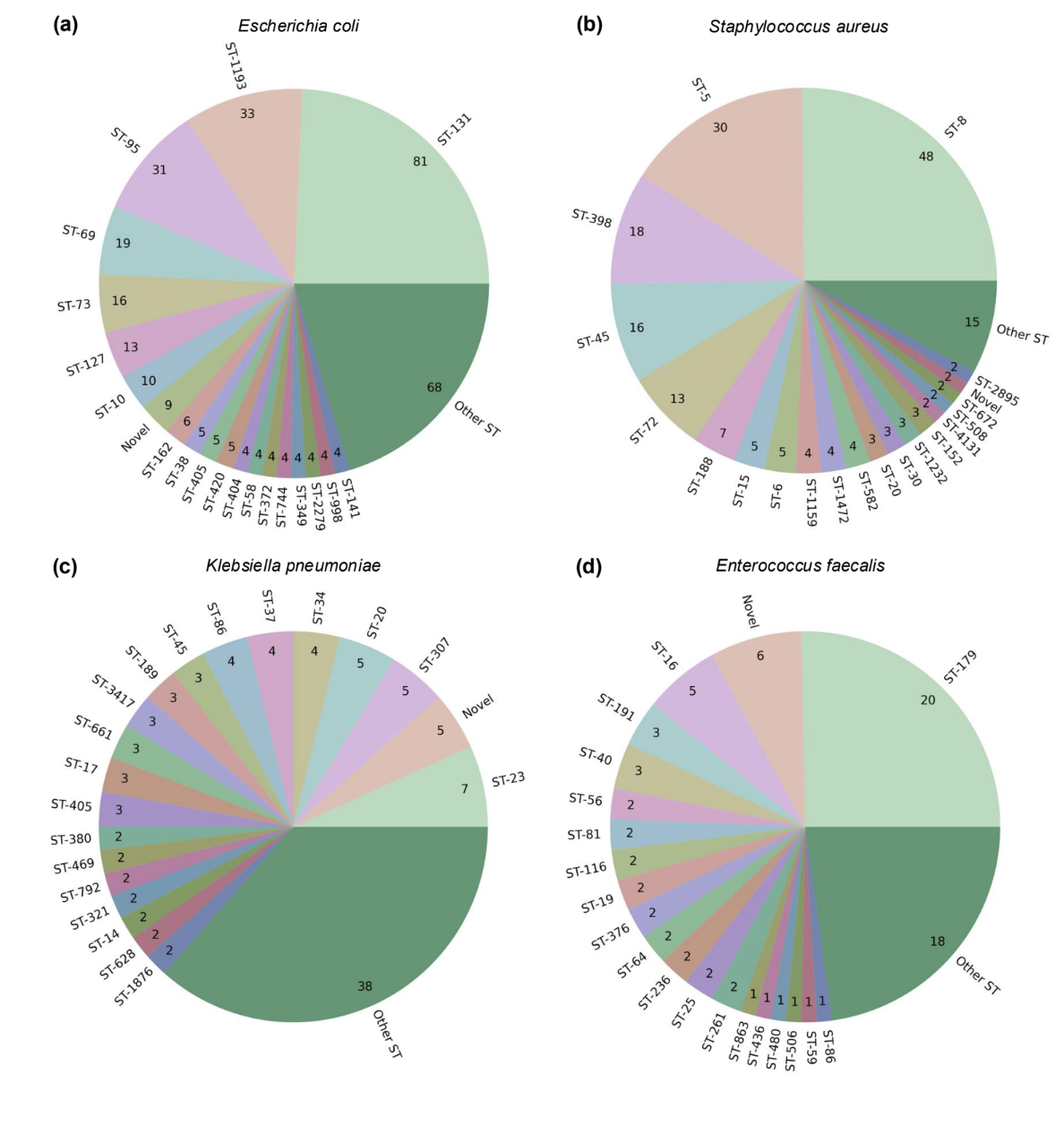


### Bibliography

[1. Antonopoulos DA, Assaf R, Aziz RK, Brettin T, Bun C, Conrad N, Davis JJ, Dietrich EM, Disz T, Gerdes S, Kenyon RW, Machi D, Mao C, Murphy-Olson DE, Nordberg EK, Olsen GJ, Olson R, Overbeek R, Parrello B, Pusch GD, Santerre J, Shukla M, Stevens RL, VanOeffelen M, Vonstein V, Warren AS, Wattam AR, Xia F, Yoo H. 2017. PATRIC as a unique resource for studying antimicrobial resistance. 4. Brief Bioinform 20:1094–1102.](https://www.zotero.org/google-docs/?mXtiRe)

[2. 2024. National Database of Antibiotic Resistant Organisms (NDARO) - Pathogen Detection - NCBI.](https://www.zotero.org/google-docs/?mXtiRe)

[3. Chen T, Guestrin C. 2016. XGBoost: A Scalable Tree Boosting System, p. 785–794. *In* Proceedings of the 22nd ACM SIGKDD International Conference on Knowledge Discovery and Data Mining. Association for Computing Machinery, New York, NY, USA.](https://www.zotero.org/google-docs/?mXtiRe)
